# Microbiota-dependent metabolite and cardiovascular disease marker trimethylamine-*N*-oxide (TMAO) is associated with monocyte activation but not platelet function in untreated HIV infection

**DOI:** 10.1186/s12879-017-2547-x

**Published:** 2017-06-23

**Authors:** Judith M. Haissman, Anna K. Haugaard, Sisse R. Ostrowski, Rolf K. Berge, Johannes R. Hov, Marius Trøseid, Susanne D. Nielsen

**Affiliations:** 1 Viro-immunology Research Unit, Department of Infectious Diseases, Rigshospitalet, University of Copenhagen, Blegdamsvej 9, 2100 Copenhagen, Denmark; 2 Department of Clinical Immunology, Capital Region Bloodbank, Rigshospitalet, University of Copenhagen, Copenhagen, Denmark; 3 Department of Clinical Science, University of Bergen, Bergen, Norway; 4 Department of Cardiology, Oslo University Hospital Ullevål, Oslo, Norway; 5 Institute of Clinical Medicine, Faculty of Medicine, University of Oslo, Oslo, Norway; 6 K.G. Jebsen Centre for Inflammation Research, University of Oslo, Oslo, Norway; 7 Research Institute of Internal Medicine, Division of Surgery, Inflammatory diseases and Transplantation, Oslo University Hospital Rikshospitalet, Oslo, Norway; 8 Norwegian PSC Research Centre, Department of Transplantation Medicine, Oslo University Hospital Rikshospitalet, Oslo, Norway; 9 Section of Gastroenterology, Oslo University Hospital Rikshospitalet, Oslo, Norway; 10 Section of Clinical Immunology and Infectious Diseases, Oslo University Hospital Rikshospitalet, Oslo, Norway

## Abstract

**Background:**

HIV infection is associated with increased risk of cardiovascular disease beyond that explained by traditional risk factors. Altered gut microbiota, microbial translocation, and immune activation have been proposed as potential triggers. The microbiota-dependent metabolite trimethylamine-*N*-oxide (TMAO) predicts myocardial infarction (MI) in the general population and has recently been shown to induce platelet hyperreactivity. In the present study, we investigated if TMAO was associated with platelet function, microbial translocation, and immune activation in both untreated and combination anti-retroviral therapy (cART) HIV infection.

**Methods:**

TMAO and the pre-cursors betaine, choline, and carnitine were quantified by mass-spectrometry in plasma samples from a previously established cross-sectional cohort of 50 untreated and 50 cART treated HIV-infected individuals. Whole-blood impedance aggregometry, C-reactive protein, sCD14, and lipopolysaccharide were assessed as measures of platelet function, inflammation, monocyte activation, and microbial translocation, respectively.

**Results:**

TMAO was not associated with platelet aggregation response after stimulation with four different agonists, or with overall hypo- or hyperreactivity in untreated or treated HIV-infected individuals. In contrast, sCD14 a marker of both monocyte activation and microbial translocation was independently associated with TMAO in untreated HIV-infection (*R* = 0.381, *P* = 0.008). Lower levels of carnitine [32.2 (28.4–36.8) vs. 38.2 (33.6–42.0), *P* = 0.001] and betaine [33.1 (27.3–43.4) vs.37.4 (31.5–48.7, *P* = 0.02], but similar TMAO levels [3.8 (2.3–6.1), vs. 2.9 μM (1.9–4.8) *P* = 0.15] were found in cART treated compared to untreated HIV-infected individuals, resulting in higher ratios of TMAO/carnitine [0.12 (0.07–0.20) vs. 0.08 (0.05–0.11), *P* = 0.02] and TMAO/betaine [0.11 (0.07–0.17) vs. 0.08 (0.05–0.13), P 0.02].

**Conclusions:**

In contrast to recent studies in HIV-uninfected populations, the present study found no evidence of TMAO-induced platelet hyperreactivity in HIV infected individuals. Microbial translocation and monocyte activation may affect TMAO levels in untreated individuals. Furthermore, the elevated ratios of TMAO/betaine and TMAO/carnitine in cART-treated individuals could possibly suggest a role of cART in TMAO metabolism.

## Background

Since the introduction of combination antiretroviral therapy (cART), AIDS and HIV-related mortality has declined in HIV-infected individuals, while cardiovascular disease (CVD) has emerged as one of the leading causes of morbidity and mortality [1, 2]. Increasing evidence indicates that HIV infection is associated with increased risk of CVD beyond that explained by the higher burden of traditional risk factors among HIV-infected individuals [3–5]. Numerous non-traditional mechanisms have been proposed including HIV-related disruption of the intestinal barrier and changes in the composition of the intestinal microbiota [6–9]. Several studies have suggested an important link between intestinal microbial composition and metabolism and the development of CVD [10–12]. In the general population, the microbiota dependent metabolite trimethylamine-*N*-oxide (TMAO) has been associated with development of clinical CVD independently of traditional CVD risk factors [13–21]. Previously, TMAO was shown to promote atherosclerosis through foam cell formation and interference with reverse cholesterol transport from the atherosclerotic plaque [13, 17, 22]. In addition, results from a recent study suggest that TMAO also induces platelet hyperreactivity [23]. Altered platelet function has been shown in HIV infection repeatedly [24–26]. We recently found TMAO to be positively associated a sub-clinical measure of coronary atherosclerosis in cART treated HIV-infected individuals [27]. However, we did not find any difference in TMAO levels in HIV-infected individuals compared to uninfected controls, but an association between TMAO and cART, especially PI use [27]. Surprisingly, TMAO was not associated with myocardial infarction (MI) in cART treated individuals in our previous study [27]. Conflicting results have also been found in the small number of other studies investigating TMAO and CVD in HIV-infected individuals, and thus the contribution of TMAO to CVD in HIV infection remains unclear [27–30].

We hypothesized that TMAO would be associated with platelet function in HIV-infected individuals. Further, as viral replication, inflammation, monocyte activation, microbial translocation, and cART have previously been linked with platelet function in HIV-infected individuals, we sought to investigate the potential influence of these factors on TMAO levels in HIV-infected individuals. To explore this hypothesis, we measured TMAO and the pre-cursors choline, carnitine, and betaine in stored plasma from untreated and treated HIV-infected individuals with previously assessed biomarkers of coagulation activity, inflammation, monocyte activation, microbial translocation and platelet function.

## Methods

### Cross-sectional cohort

The cohort included 50 HIV-infected, untreated individuals, from the Department of Infectious Diseases, Copenhagen University Hospital, and 50 HIV-infected individuals on cART, selected to match the untreated group for age, gender, and current CD4^+^ T-cell count. The cohort has previously been described in detail [31]. One individual in the treatment group had detectable viral replication and was excluded from further analyses. Clinical characteristics of the study population have previously been described [31]. Briefly, mean age was 40 and 42 years, 90 and 88% were men, 90 and 86% were Caucasian, 42 and 41% were current smokers, and mean current CD4^+^ T-cell count was 600 and 674 in the HIV-infected untreated and treated individuals, respectively [31]. Baseline characteristics are summarized in Table 1.

**Table 1:** Clinical characteristics of the study cohort

	Untreated (*n* = 50)	ART-Treated (*n* = 49)	*P* value
Age, years	41 (33–46)	43 (36–48)	0.249
Sex	90% (45) Male	88% (43) Male	0.722
Etnicity	90% (45) Caucasian	86% (42) Caucasian	0.514
Transmission of HIV	82% (41) MSM	74% (36) MSM	0.618
Current smoker	42% (21)	41% (20)	0.869
HIV-RNA, (copies/mL)	23,026 (5517–90,321)	19 (19–20)	< 0.001
Current CD4^+^ T-cell count, (cells/μL)	560 (415–795)	610 (480–875)	0.363
Duration of HIV^a^ (months)	28 (8–75)	73 (36–151)	0.009

Summary of clinical and demographic characteristics of the study population previously published in [31]. from Continuous data are presented as medians and (interquartile ranges) and categorical data as percentages and (total numbers). Untreated and treated individuals were compared by Mann-Whitney U test

^a^Duration since 1^st^ positive HIV-1 test

*cART* antiretroviral therapy, *MSM* Men who have sex with men

Platelet function was determined at inclusion by whole-blood multiple electrode impedance aggregometry, assessing platelet aggregation in a time-dependent manner as area under curve after 6 min after stimulation with adenosine diphosphate (ADP, concentration 6.5 mmol/l, arachidonic acid (ASPI, concentration 0.5 mmol/l), collagen (COL, 3.2 mg/ml), and thrombin receptor agonist peptide (TRAP, concentration 32 mmol/l) on a Multiplate analyzer (Dynabyte GmBH, Munich, Germany), as previously described [31].

Routine biochemistry including standard hemostatic whole blood tests, and assessment of inflammation, monocyte activation and microbial translocation with C-reactive protein (CRP), soluble CD14 (sCD14), and lipopolysaccharide (LPS) by quantification with enzyme linked immunosorbent assay (ELISA) and Limulus amebocyte lysate (LAL) test for LPS, respectively, have previously been performed [31, 32].

### Ethics and informed consent

Plasma samples collected from all participants were stored at −80 °C until analysis.

The study was conducted in accordance with the Helsinki-declaration and approved by the Committee on Biomedical Research Ethics in Denmark (H-2-2009-089) and the Danish Data Protection Agency. Written informed consent including consent to store plasma and perform further analysis on blood samples was obtained from all participants after oral and written information.

### Measurement of TMAO

Stable-isotope dilution liquid chromatography with tandem mass spectrometry was used for quantification of TMAO and pre-cursors choline, carnitine and betaine as previously described [16, 33]. Valid measurements were obtained from all samples for betaine, choline and carnitine. For TMAO measurements, one sample was a clear outlier with a result of 52.5 μM, which is much higher than the 98-percentile of 12.9 μM and therefore excluded from further analyses.

### Statistical analyses

Data are given as median and interquartile range (IQR). Levels of TMAO and pre-cursors in untreated and treated groups were compared using Student’s t test after natural logarithmic (ln) transformation to obtain normal distribution. Univariate linear regression was performed to investigate possible association with TMAO. Residuals were checked for normal distribution and ln transformation was performed when appropriate. Data are given as standardized residuals. Significant univariate associations were investigated in a multivariate regression model. Independent variables included sCD14, age and gender as the sample-size was small. Models including a fifth variable were created adding one variable at the time. A *P-*value < 0.05 was considered statistically significant. Analysis was performed using SPSS 19 (IBM Inc., Armonk, NY).

## Results

### TMAO was not associated with platelet aggregation

No associations were found between TMAO and standard coagulation markers i.e. D-dimer, fibrinogen, activated partial tromboplastin time (APTT), coagulation factors II-VII-X, and platelet count (Table 2). In addition, no associations were found between TMAO and platelet function evaluated as platelets aggregation response to ADP, ASPI, COL and TRAP (Table 2). This was consistent in both untreated and treated HIV-infected individuals, and platelet aggregation was evaluated both as a continuous variable and categorically classified as hyper/hypocoagulable (Table 2). Regression coefficients and *p*-values are given in Table 2.

**Table 2:** Univariate linear regression with TMAO as the dependent variable

	Untreated (*n* = 49)		ART Treated (*n* = 49)	
	Standardized β coefficients	*P*	Standardized β coefficients	*P*
Clinical characteristics:				
Age (years)	0.180	0.21	0.290	0.04
Gender, male	0.137	0.34	0.110	0.94
Current smoker,	0.129	0.39	−0.182	0.24
Current CD4+ T cell count (cells/μl)	0.035	0.81	0.084	0.57
Nadir CD4+ T cell count (cells/μl)	−0.081	0.58	0.087	0.55
HIV-RNA (10^3^ copies/ml)	−0.010	0.94	*NA*	*NA*
HIV duration	0.078	0.59	0.070	0.64
Class of ART				
NRTI containing (*n* = 44)	*NA*	*NA*	0.043	0.78
NNRTI containing (*n* = 22)	*NA*	*NA*	0.135	0.38
PI containing (*n* = 23)	*NA*	*NA*	0.145	0.34
II containing (*n* = 4)	*NA*	*NA*	−0.024	0.87
Abacavir containing (*n* = 16)	*NA*	*NA*	−0.126	0.41
Standard coagulation				
D-dimer	0.051	0.74	0.209	0.16
Fibrinogen	0.091	0.55	0.183	0.24
APTT	0.117	0.43	0.171	0.27
Coagulation factors 2–7-10	0.043	0.77	0.032	0.83
Platelet aggregation (Multiplate):				
ADP test (Units)	−0.074	0.62	−0.177	0.23
ASPI test (Units)	−0.156	0.29	−0.243	0.10
COL test (Units)	−0.080	0.59	−0.211	0.15
TRAP test (Units)	−0.119	0.42	−0.220	0.13
Hypocoagulable in ≥2/4 tests	0.007	0.97	0.147	0.32
Hypercoagulable in ≥2/4 tests	−0.172	0.24	*NA*	*NA*
Markers of Microbial translocation and inflammation:				
sCD14 (ρg/ml)	**0.454**	**0.001**	−0.157	0.28
LPS (ρg/ml)	−0.019	0.90	0.047	0.75
hsCRP	−0.016	0.92	0.028	0.37

Univariate linear regression with trimethylamine-N-oxide (TMAO) as the dependent variable. Standardized regression coefficients and *P* values are given. Standard coagulation, and platelet impedance aggregometry are given as continuous variables and classified as hypo- and hypercoagulable according to normal range

*APTT* activated partial thromboplastin time, *ART* anti-retroviral therapy, *ASPI* arachidonic acid, *COL* collagen, *FEU* fibrinogen equivalent units, *II* integrase Inhibitor, *n* number, *NA* not applicable, *NRTI* nucleotide/nucleoside reverse transcription inhibitor, *NNRTI* non-nucleoside reverse transcription inhibitor, *PI* protease Inhibitor, *sCD14* soluble CD14, *TRAP* thrombin-receptor activating peptide

Significant associations are marked in bold

### sCD14 was an independent predictor of TMAO in untreated HIV infection

A positive association was found between TMAO and sCD14 in untreated HIV-infected individuals, but not in treated HIV-infected individuals (Table 2). No associations between TMAO and LPS or CRP were found (Table 2). In multivariate linear regression models, sCD14 remained an independent predictor of TMAO after adjustment for age, gender, and smoking (Table 3). A fifth variable (viral load, CD4^+^ T-cell count, LPS, or CRP) was added to the model one at the time, and sCD14 remained an independent predictor of TMAO in untreated HIV-infected individuals with each added variable (Table 3).

**Table 3:** Multiple Linear Regression Models with TMAO as Dependent Variable

Characteristics	Standardized β coefficient	*P*
Age	0.233	0.10
Gender	0.079	0.58
Smoking	0.123	0.39
sCD14	0.381	0.008
Model with additional adjustment for each of the following variables		
CD4^+^ T-cell count	0.379	0.010
Viral load	0.452	0.003
hsCRP	0.304	0.045
LPS	0.384	0.009

Multivariate linear regression analyses with TMAO as dependent variable. Standardized β coefficients for TMAO are given after adjustment for a fifth variable. sCD14, soluble CD14, TMAO, Trimethylamine-*N*-oxide

### TMAO was not associated with HIV-related factors

TMAO was not associated with CD4^+^ T-cell count, nadir CD4^+^ T-cell count, HIV duration or viral load in untreated HIV-infected individuals, or with CD4^+^ T-cell count, nadir CD4^+^ T-cell count or HIV duration in treated HIV-infected individuals (Table 2). Neither was TMAO associated with cART regimens (Table 2). No associations between TMAO and gender or smoking status were found in either untreated or treated HIV-infected individuals (Table 2). A weak positive association was found between TMAO and age in treated HIV-infected individuals. The association with age was not found in untreated individuals.

### TMAO and precursors in untreated vs. cART treated HIV-infected individuals

**Fig. 1:**
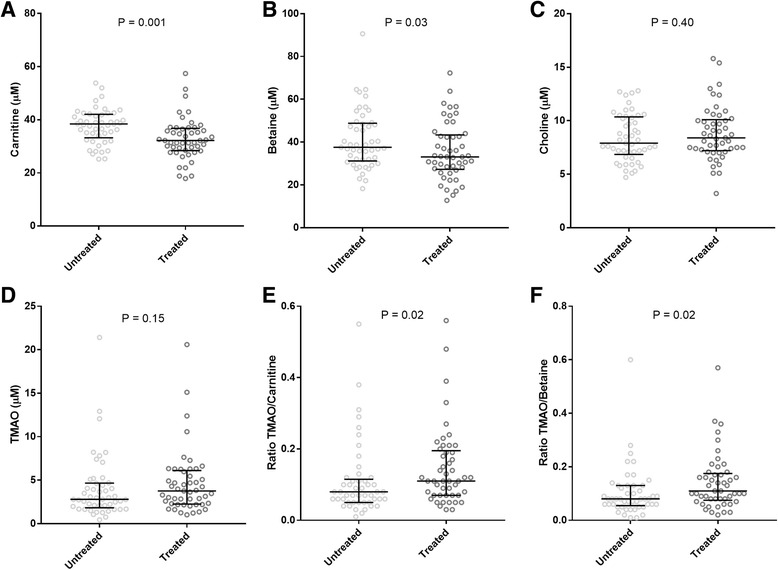
Trimethylamine-N-oxide (TMAO), pre-cursors and TMAO/pre-cursor ratios in untreated compared to cART treated individuals. Comparison of untreated (n = 49) and treated (n = 49) HIV-infected individuals of levels of Carnitine (**a**), Betaine (**b**), Choline (**c**), and TMAO (**d**), and ratios of TMAO/Carnitine (**e**), TMAO/Betaine (**f**) For each group median and interquartile ranges are shown. Data was ln-transformed and student t-test was used to compare groups, and *P*-values are given for each comparison

TMAO levels were not significantly different in untreated vs. treated HIV-infected individuals [2.9 μM (1.9–4.8) vs. 3.8 (2.3–6.1), *P* = 0.15] (Fig.1d). However, elevated carnitine [38.2 (33.6–42.0) vs. 32.2 (28.4–36.8), *P* = 0.001] and betaine [37.4 (31.5–48.7 vs. 33.1 (27.3–43.4), *P* = 0.02], but not choline [7.9 (6.8–10.3) vs. 8.4 (7.2–10.1), *P* = 0.40] were found in untreated compared to treated HIV-infected individuals (Fig. 1a, 1b, 1c). This resulted in elevated ratios of TMAO/carnitine [0.12 (0.07–0.20) vs. 0.08 (0.05–0.11), *P* = 0.02] (Fig. 1e) and TMAO/betaine [0.11 (0.07–0.17) vs. 0.08 (0.05–0.13), P 0.02] (Fig. 1f), but not TMAO/choline [0.45 (0.31–0.69) vs. 0.35 (0.26–0.55), *P* = 0.25] in treated compared to untreated HIV-infected individuals. Elevated ratios remained after adjusting for sCD14 and viral load using multivariate analysis of variance (ANOVA) (data not shown).

## Discussion

In this study including 50 untreated and 50 cART treated HIV-infected individuals no associations were found between TMAO and platelet function. In contrast, TMAO was independently associated with sCD14 in untreated but not in treated HIV-infected individuals. Furthermore, significantly elevated ratios of TMAO over pre-cursors were found in treated compared to untreated HIV-infection.

In the general population, there is increasing appreciation that changes in the composition and function of the gut microbiota can promote long-term susceptibility to CVD [34]. The gut microbe-derived metabolite TMAO has been recognized as an important contributor in this process, and recent evidence points towards TMAO induced platelet hyperreactivity as a potential pathogenic mechanism [23]. We were not able to confirm this finding in an HIV-infected cohort. The explanation of the lacking association between TMAO and platelet function could be, that other factors have a stronger effect on platelet function in HIV-infected individuals than TMAO, thus diluting the direct effect of TMAO on platelet function. Indeed, previous studies in HIV-infected populations indicate that both immune activation, viral replication, specific cART drug classes lead to platelet activation and dysfunction [24, 31, 35, 36].

Further, some of these factors might potentially interfere with TMAO levels. Indeed, a strong association was found between sCD14 and TMAO in untreated HIV-infected individuals. This association remained significant after adjustment for demographic and HIV-related factors. sCD14 is secreted from activated monocytes upon binding of the microbial product LPS to the toll like receptor (TLR)-4 [37, 38]. Thus, sCD14 can be used as a marker of both monocyte activation and microbial translocation. Recent evidence suggests that microbial translocation is accompanied by alterations in the composition of the gastrointestinal microbiota [6–8, 39]. Hence, the association between sCD14 and TMAO in our study could imply that a gut microbiota composition associated with microbial translocation might be of a phenotype that produce higher levels of TMAO. To our knowledge, no previous studies have found a link between microbial translocation, monocyte activation, and TMAO. However, associations between TMAO and systemic inflammatory marker CRP or microbial translocation marker LPS were not found. Both markers are known to be volatile, and previous studies have reported lacking or weak associations between LPS and CRP and other markers of microbial translocation and systemic inflammation [40–43]. Further, interpretation of the association between sCD14 and TMAO as proof of the impact of microbial translocation on plasma TMAO cannot be inferred. CD14 is also a co-receptor for other TLRs and microbial products other than LPS. Thus, elevated sCD14 is not strictly induced by microbial translocation and therefore also a marker of more general monocyte activation.

Elevated betaine and carnitine were found in untreated compared to treated HIV-infected individuals. Both the endogenously produced metabolite betaine and the dietary metabolite carnitine can be metabolized to TMAO by gut microbiota [12, 18]. Both pre-cursors have also been associated with CVD [44], although a recent study suggests that this association is mediated through the concomitant increase in TMAO [18]. As we do not have any information on dietary intake, an effect of diet on carnitine levels, particularly increased intake of red meat, cannot be excluded. However, the increased ratios of TMAO over both endogenous and diet dependent pre-cursors in cART treated individuals suggest the involvement of other mechanisms. We and others have previously found elevated TMAO associated with cART treatment, especially PI-treatment [27, 45]. The elevated ratios of TMAO over pre-cursors in cART treated individuals may suggest that cART interferes with TMAO metabolism, possibly by inducing hepatic flavin-containing monooxygenases resulting in increased conversion from TMA to TMAO. Commencement of cART therapy leads to several other changes including suppression of viral replication and concomitant attenuation of immune activation. Thus, changes in these factors could also be the cause of the observed changes in TMAO production and metabolism observed in the study. However, even after adjusting for viral replication and monocyte activation increased ratios of TMAO over precursors betaine and carnitine remained in cART treated individuals. These results offer a possible explanation for the lacking power of TMAO in predicting MI in cART treated HIV-infected individuals [27], and for the association between TMAO and sCD14 found in only untreated and not cART treated individuals in this study. However, as the study populations of untreated and cART treated HIV-infected infected individuals differ on several important known and unknown factors e.g. HIV duration, illicit drug use, socioeconomic status, dietary habits. These additional factors might also account for the elevated ratios and pre-cursors in cART treated individuals.

The study was limited by the cross-sectional design, rendering conclusions on causality unanswered. In contrast to previous results [27, 45], no association of TMAO and PI use was found. However, this might be due to the small number of participants on different ART regimens (*n* = 23, PI), increasing the risk of type two statistical errors. Lacking power due to limited number of participants and high variance in TMAO concentrations, might also explain why the elevation of TMAO in treated compared to untreated individuals did not reach statistical significance. Methodology for assessing platelet function differed from the study that found TMAO induced hyperreactivity in platelets from HIV-uninfected individuals [23]. Platelet aggregation was assessed by measuring changes of impedance in electrodes after stimulation with four different stimulants including ADP (Multiple electrode aggregation, Multiplate) [31]. In the study by Zhu et al. aggregation was assessed by changes in light transmission after stimulation with ADP and thrombin (light transmission aggregation, LTA) [23]. Even though one of the same stimulants was used in both studies, the slight differences in methodology might account for the different findings, and concentrations of stimulants cannot be directly compared [46]. Further, impedance aggregometry utilizes whole-blood samples without prior manipulation, rendering the method closer to the biological environment compared to the platelet rich plasma utilized in LTA, where platelets prior to use have been centrifuged possibly inducing activation or damage. Additional methods for evaluating platelet function and an HIV-uninfected control group could have helped distinguish between specific HIV-related effects and missing associations due to methodical issues. Furthermore, additional markers of immune activation independent of microbial translocation, other markers specifically assessing microbial translocation in addition to LPS, and an assessment of the microbial composition of the study participants would have facilitated the exploration of the specific effects of immune activation in opposition to microbial translocation and composition on TMAO levels. Finally, the analyses were conducted on a previously established study population.

## Conclusions

In conclusion, no evidence of TMAO being associated with platelet hyperreactivity was found in an HIV-infected study population. This result is in contradiction to findings from a study in HIV-uninfected individuals. Interestingly, TMAO was independently associated with sCD14 in untreated HIV-infected individuals, suggesting that microbial translocation and monocyte activation may affect TMAO levels. Furthermore, the elevated ratios of TMAO/betaine and TMAO/carnitine in cART-treated individuals suggest an altered TMAO metabolism in cART treated-individuals. Our findings call for further studies in HIV-infected populations specifically designed to investigate the potential role of gut microbiota composition and related metabolites, beyond TMAO, on CVD risk. Furthermore, the elevated ratios of TMAO/betaine and TMAO/carnitine in cART-treated individuals suggest an altered TMAO metabolism in cART treated-individuals. This is also an important avenue of future research to explore, as the altered metabolism suggested from these and previous findings, might have an impact on CVD risk in cART treated HIV-infected individuals.

## Abbreviations

ADP: Adenosine diphosphate
AIDS: Acquired immunodeficiency syndrome
ANOVA: Analysis of variance
APTT: Activated partial thromboplastin time
ASPI: Arachidonic acid
cART: Combination antiretroviral therapy
CRP: C-reactive protein
CVD: Cardiovascular disease
ELISA: Enzyme linked immunosorbent assay
FEU: Fibrinogen equivalent units
HIV: Human immunodeficiency virus
II: Integrase Inhibitor
IQR: Interquartile range
LAL: Limulus amoebocyte lysate
Ln: Natural logarithmic
LPS: Lipopolysaccharide
LTA: Light transmission aggregation
MI: Myocardial infarction
N: Number
NA: Not applicable
NNRTI: Non-nucleoside reverse transcription inhibitor
NRTI: Nucleotide/nucleoside reverse transcription inhibitor
PI: Protease Inhibitor
sCD14: Soluble CD14
TLR: Toll like receptor
TMAO: Trimethylamine-*N*-oxide
TRAP: Thrombin receptor agonist peptide
